# Improved Method for Electron Powder Diffraction-Based Rietveld Analysis of Nanomaterials

**DOI:** 10.3390/nano14050444

**Published:** 2024-02-28

**Authors:** Viktória K. Kis, Zsolt Kovács, Zsolt Czigány

**Affiliations:** 1 HUN-REN Centre for Energy Research, Institute of Technical Physics and Materials Science, Konkoly-Thege Miklós út 29-33, H-1121 Budapest, Hungary; 2 Department of Mineralogy, Eötvös Loránd University, Pázmány Péter sétány 1/c, H-1117 Budapest, Hungary; 3 Department of Materials Physics, Eötvös Loránd University, Pázmány Péter sétány 1/a, H-1117 Budapest, Hungary; kovacs.zsolt@ttk.elte.hu

**Keywords:** electron diffraction, nanostructure characterization, instrumental broadening, Rietveld analysis, nanopowder

## Abstract

Multiphase nanomaterials are of increasing importance in material science. Providing reliable and statistically meaningful information on their average nanostructure is essential for synthesis control and applications. In this paper, we propose a novel procedure that simplifies and makes more effective the electron powder diffraction-based Rietveld analysis of nanomaterials. Our single step in-TEM method allows to obtain the instrumental broadening function of the TEM directly from a single measurement without the need for an additional X-ray diffraction measurement. Using a multilayer graphene calibration standard and applying properly controlled acquisition conditions on a spherical aberration-corrected microscope, we achieved the instrumental broadening of ±0.01 Å in terms of interplanar spacing. The shape of the diffraction peaks is modeled as a function of the scattering angle using the Caglioti relation, and the obtained parameters for instrumental broadening can be directly applied in the Rietveld analysis of electron diffraction data of the analyzed specimen. During peak shape analysis, the instrumental broadening parameters of the TEM are controlled separately from nanostructure-related peak broadening effects, which contribute to the higher reliability of nanostructure information extracted from electron diffraction patterns. The potential of the proposed procedure is demonstrated through the Rietveld analysis of hematite nanopowder and two-component Cu-Ni nanocrystalline thin film specimens.

## 1. Introduction

Nanostructured materials are present in our everyday life, both of anthropogenic origin and in their natural form. Nanoparticles have a wide range of technological applications, e.g., as catalytic agents, optoelectronic devices, or active ingredients in pharmaceuticals [1]. Bulk nanomaterials comprise multicomponent melt-spun metallic alloys, inherently nanostructured thin films, or nanocomposites. As the industrial production and application of nanomaterials increases, an increasing amount is released into the environment, which accumulating in natural water bodies or in soil may trigger harmful effects on human health [2]. The performance of nanomaterials, including their desired application and unintentional or adverse effects, is controlled by their atomic structure, particle size, and phase composition.

Electron diffraction is an ideal tool for the characterization of nanomaterials. In comparison with other diffraction techniques like X-ray or synchrotron diffraction, the main advantage of electron diffraction is their higher efficiency when detecting light elements and their locality. Selected area electron diffraction (SAED) measurements can be performed on nanocrystalline thin film areas even below 100 nm of the diameter or using extremely small quantities of nanopowder (electron powder diffraction, EPD) and provide sufficient signal and excellent signal-to-noise ratio for quantitative analysis on the second timescale. The reproducibility and accuracy of electron diffraction can be compatible with those of routine laboratory X-ray diffraction (XRD) measurements, provided that acquisition parameters are properly controlled and the resulting SAED patterns are calibrated [3]. Additionally, EPD or SAED is complementary to other local transmission electron microscopy (TEM)-based characterization techniques like high-resolution transmission electron microscopy (HRTEM) or nanobeam diffraction because they provide nanostructure information of statistical relevance.

The quantitative evaluation of powder diffraction patterns relies on the accurate measurement of diffraction peak intensities. In the case of complex structures or multicomponent materials, peaks frequently overlap, which makes the extraction of intensity values uncertain. Whole pattern fitting methods aim to overcome this problem using all the diffraction information in the measured scattering angle range. Rietveld analysis [4] is an established method of the whole pattern fitting of neutron and X-ray powder diffraction patterns, which provides quantitative data on phase composition, crystal structure, crystallite size, shape, preferred orientation, etc. (a comprehensive list of software toolkits performing Rietveld analysis can be found on the IUCr website https://www.iucr.org/resources/other-directories/software (accessed on 26 February 2024). Microstructure information is included in the diffraction line profile, i.e., the shape and broadening of the diffraction peaks; however, peak broadening also exhibits an instrumental broadening effect, which is related to the applied experimental setup. Thus, to obtain microstructure information from Rietveld analysis, it is of fundamental importance to properly separate the scattering angle-dependent sample and instrumental contributions to peak broadening, *f*(*Q*) and *g*(*Q*), respectively.

The Rietveld method is used less for the evaluation of electron diffraction patterns obtained from nanomaterials (SAED or EPD), mostly because of the potentially high contribution of multiple scattered electrons [5] and also because of the large influence of the setting of electron optics on the variation in detected intensity. The effect of multiple scattering on diffraction peak intensities as a function of the average atomic number and crystal thickness has been analyzed in the published literature in detail [5]. In nanocrystalline materials, in general, it is much weaker than single crystal samples [6]. In diffraction measurements performed on small crystallites at high enough electron energies, the kinematical scattering of electrons dominates [7]. Also, in the case of lower symmetry crystals, the probability of multiple scattering is reduced [8]. The predominance of kinematical scattering in the case of anatase TiO_2_ nanoparticles of an average size of 7 nm at 120 keV was proven by calculations [9]. For MnFe_2_O_4_ nanoparticles, experimental structure factor amplitudes obtained from the Rietveld refinement of SAED patterns were compared with calculated kinematical and dynamical amplitudes, and it was found that at about 7 nm, crystallite thickness at 120 keV kinematical conditions was expected [10]. In the case of Au_3_Fe_1−x_ alloy nanoparticles of 9.5 nm thickness, kinematical intensities at 200 keV were obtained by applying an approximately 2.5% correction [11] based on the Blackman two-beam approximation theory. These results indicate that the dominance of the kinematical scattering of 200 keV electrons is a reasonable presumption when the crystallite size of nanoparticles is close to 10 nm, and no significant overlapping of particles occurs.

The other issue limiting the applicability of the whole pattern fitting of ring-like electron diffraction patterns is related to instrumental broadening. Early works on electron diffraction-based Rietveld analysis reported full width at half maximum (FWHM) values extracted from the analysis but did not demonstrate the difference between the instrumental and sample contribution to diffraction peak broadening [7,12]. In Ref. [7], the authors highlight that diffraction profiles obtained from unfiltered electron diffraction patterns exhibit 10–20% larger FWHM values than corresponding energy-filtered patterns. They also point out that the Rietveld method provides accurate lattice parameters and yields correct refined atomic coordinates even from non-energy-filtered data. However, in their subsequent work using energy-filtered electron powder diffraction data [9], no attempt was made to assess the crystallite size from diffraction peak broadening. In general, publications focus on the determination of the crystal structure [10,13], lattice parameter [11,12], occupancy change as a function of temperature [11] or texture analysis [14]. These publications reported the average particle size obtained using alternative techniques like bright or dark field as well as HRTEM imaging, and in these works, no attention was paid to the nanostructural information hidden in peak broadening. However, in their paper, Ref. [13] considers peak broadening and applied FWHM values to quantify the data quality difference between electron diffraction patterns recorded using different techniques like conventional, precession, and theta-scan precession electron diffraction.

Boullay et al. [15] extended the quantitative analysis of electron diffraction patterns to nanostructural properties for the first time. They studied rutile and hausmannite nanoparticles and extracted the average size and shape data of the crystallites from ring-like electron diffraction patterns. To perform this, they established a two-step calibration procedure for modeling the instrumental component of peak shape broadening in the TEM. This procedure allowed the latter to separate successfully the scattering contribution of ZnO and ZnS in a two-phase nanocrystalline powder using electron diffraction data [16]. The methodology of the two-step calibration procedure is detailed, and the effect of different TEM operation conditions is also discussed in a recent publication [17].

The procedure proposed for the determination of the instrumental broadening of the TEM in [15] is the following. As an initial step, the instrumental broadening of an X-ray diffractometer as a function of the scattering vector Q (Q = 4π sin θ/λ), $g_{XR}(Q)$ has to be determined using a sample of a high degree of crystallinity as the calibration standard. Then, a nanocrystalline calibration standard suitable for both XRD and electron diffraction is selected, preferably a stable oxide nanopowder of uniform and isometric size in the range of 10–20 nm. Using this nanopowder calibration standard, the sample contribution ($f_{XR}\left(Q\right)$) to the measured profile ($h_{XR}(Q)$) is obtained as a convolution of $g_{XR}(Q)$ and $f_{XR}\left(Q\right)$ according to the following relation:

(1) $$h_{XR}(Q)=g_{XR}(Q)⊗f_{XR}\left(Q\right)+b_{XR}(Q)$$

where $b_{XR}\left(Q\right)$ is the background function. Then, the same sample is measured by EPD. As the sample contribution $f_{XR}\left(Q\right)$ of the nanopowder calibration sample is known, it is used as input data during the Rietveld analysis of EDP. This step allows $g_{TEM}(Q)$, the instrumental broadening function of the TEM, to be determined according to the following relation:

(2) $$h_{TEM}(Q)=g_{TEM}(Q)⊗f_{XR}\left(Q\right)+b_{TEM}(Q)$$

where $h_{TEM}(Q)$ is the intensity profile obtained from the ring-like electron diffraction pattern by summing up intensities at the same angular distance from the direct beam and $b_{TEM}\left(Q\right)$ is the background function of the electron diffraction pattern.

According to this procedure, the determination of the instrumental broadening of a TEM requires three independent measurements and two calibration samples. As an alternative approach, in this work, we present a single step in TEM procedure, which allows the instrumental broadening function of TEM to be obtained using a multilayer graphene calibration sample by a single SAED measurement. This procedure is based on our previous work on SAED calibration [3], exploiting its reproducibility, the achievable ±0.1% absolute accuracy of SAED measurements, and the minimization of instrumental broadening. The potential of this procedure is demonstrated on nanopowder and nanocrystalline thin film specimens.

## 2. Materials and Methods

### 2.1. Materials

To determine instrumental broadening, electron diffraction measurements were carried out on Pelco^®^ graphene TEM support films (Ted Pella, Redding, CA, USA product # 21740) suspended on a lacey carbon-coated copper grid with a mesh size of 300. SAED patterns were recorded from several positions inside the same grid, and the pattern providing the most continuous and pure diffraction rings was selected for further analysis.

Nanocrystalline hematite was synthesized for plant nutrition experiments as detailed for sample S0 in Ref. [18]. The nanoparticle concentration of the resulting suspension was increased by a factor of four by evaporation in a vacuum. This gentle method prevents the particle’s aggregation and preserves the stability of the colloid sample. For TEM analysis, a drop of the suspension was deposited onto a lacey carbon-supported ultrathin carbon-film-covered Cu grid (Ted Pella, Redding, CA, USA). Crystallite size distribution was determined by analyzing TEM images using ImageJ 1.53k software.

**Figure 1:**
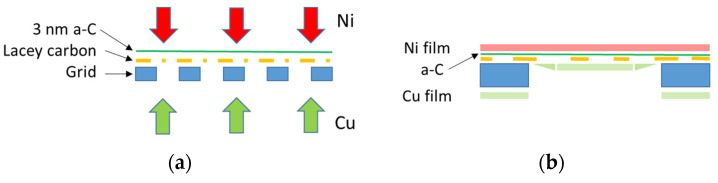
(**a**) Schematic cross-section of the TEM grid covered by a 3 nm thick ultrathin carbon foil (Ted Pella, Redding, CA, USA) showing the experimental setup during the deposition of Cu-Ni thin films. (**b**) One mesh cross-section was enlarged, indicating the possible non-uniform thickness of Cu film sputtered from the grid side due to the shadowing effect of the grid bar.

Cu-Ni thin film samples were deposited in a high vacuum system by direct current magnetron sputtering onto an ultrathin carbon-film-coated lacey carbon film supported by a 400 mesh copper grid (Ted Pella, Redding, CA, USA). The nominal thickness of the ultrathin amorphous carbon (a-C) film was 3 nm. The copper and nickel thin films were prepared by DC magnetron sputtering in an ultra-high vacuum (UHV) compatible vacuum chamber (with a base pressure of 6 × 10^−6^ Pa) in 0.3 Pa Ar with a 3 Å/s deposition rate. The power and the deposition time were selected so that the thickness of the deposited films equaled ca. 15 nm each, resulting in an overall Cu-Ni film thickness of 30 nm. To avoid epitaxial growth and strain-related lattice parameter variation at the interface, Cu and Ni were deposited onto opposite sides of the a-C foil (Figure 1). This arrangement implies that due to the shadowing effect of the grid bars during Cu deposition, the film thickness and, thus, overall composition were not uniform inside the mesh. To overcome such local compositional changes, EDS analysis and corresponding SAED patterns were taken from the same area.

### 2.2. Electron Diffraction

Electron diffraction measurements were carried out using a Themis (Thermo Fisher Scientific, Waltham, MA, USA) TEM with Cs correction in the imaging system (spatial resolution in HRTEM mode 0.8 Å), operating at 200 Kv, and equipped with a Schottky field emission gun (FEG) with an energy spread of ca. 0.7 Ev, and a four-segment Super-X EDS detector. SAED patterns were taken from an area of ca. 3 μm in diameter and recorded using a Ceta camera using Velox software (https://veloxusa.com/about-us, accessed on 19 February 2024) (Thermo Fischer Scientific, Waltham, MA, USA). SAED patterns were recorded using the highest (4 k × 4 k) camera resolution with special care of avoiding saturation and keeping the intensity in the linear range of the detector. SAED patterns were taken as detailed in [3]. Measurements were carried out at a 650 mm nominal camera length, which provided scattered intensity at a large enough angular range for Rietveld refinement. The camera length was calibrated using 30 nm thick DC-sputtered nanocrystalline Cu thin film. Care was taken to ensure standardized illumination conditions by controlling Wehnelt bias (GunLens parameter in the Themis microscope (Thermo Fischer Scientific, Waltham, MA, USA)), spot size, and C2 (second condenser lens) current during diffraction measurements. In this way, beam convergence was controlled, which is a prerequisite for the reproducible determination of the instrumental component of peak broadening at a given combination of lens currents. To avoid unexpected hysteresis effects, switching between imaging and diffraction modes, was done always at the same (45kx) magnification. After setting up the desired illumination conditions, the focusing of the diffraction pattern was performed by adjusting the diffraction lens current. Overall control on standard experimental conditions during subsequent measurements is ensured by keeping the lens currents constant better than 3 × 10^−4^.

### 2.3. Diffraction Data Pre-Processing and Rietveld Analysis

The intensity profiles of the SAED patterns were obtained by integrating intensities at the same angular distance from the direct beam using the Process Diffraction v12.0.8 software [19]. Center (x,y) and elliptical distortion (orientation α and measure of ellipticity ε) were first adjusted manually and then refined using the automated algorithm implemented in Process Diffraction for faint diffuse diffraction peaks [20]. In this evaluation approach, microscope lens distortions were parametrized as elliptical distortion, and higher-order distortions were neglected (note that the treatment of higher-order distortions is not implemented in currently available electron diffraction processing software). The FWHM values of the diffraction peaks were determined by fitting the pseudo-Voigt function [5] using Origin 2023b (10.05) software. Rietveld analysis was carried out using MAUD 2.99 software [21,22] with atomic scattering factors for electrons [23]. Although two-dimensional diffraction data were implemented in MAUD [24], we chose to use integrated intensity profiles as input data. In this way, the effects of microscope alignment and data pre-processing, i.e., camera length, center, and ellipticity, were handled separately from sample features.

## 3. Results

### 3.1. Experimental Determination of Instrumental Broadening of TEM

The broadening of the diffraction peak results from the convolution of broadening caused by beam properties, lens aberrations, and sample properties. As the peak shape contains microstructural information, to obtain quantitative data on crystallite size, anisotropic shape, or strain, the separation of instrumental and sample contributions at the peak shape is essential. In XRD, this procedure is performed using a reference sample (e.g., LaB_6_, Al_2_O_3_, or silicon) that has a well-known and defect-free crystal structure and a large enough crystallite size. Following the same concept as in XRD, for the single-step determination of the instrumental broadening of TEM, $g_{TEM}\left(Q\right)$, such a reference material is needed, which lacks sample-related broadening effects. Because of the strong interaction of electrons with matter, the thickness of the reference sample should not exceed the elastic mean free path for electrons to avoid multiple scattering. Moreover, the ideal reference sample has a smooth surface and uniform thickness and covers the TEM grid evenly. In this case, the two-step procedure for obtaining $g_{TEM}\left(Q\right)$ [15] can be simplified to the following:

(3) $$h_{TEM}\left(Q\right)=g_{TEM}\left(Q\right)⊗f_{TEM}\left(Q\right)+b_{TEM}\left(Q\right),$$

where $f_{TEM}\left(Q\right)$ is the sample contribution to the $h_{TEM}\left(Q\right)$-measured peak profile obtained by electron diffraction, and $b_{TEM}\left(Q\right)$ is the background of electron scattering. In our approach, graphene is considered as an ideal, defect-free two-dimensional crystal with lateral dimensions on the micrometer scale. Indeed, during the SAED measurement, the crystallite size of graphene is limited by the applied SA aperture, which, in our measurements, is ca. 3 μm and allows the crystallite size-related peak broadening to be removed. Due to the especially thin nature of graphene, diffraction spots are strongly elongated parallel to the incident beam, which allows the diffracted intensity to be measured at high scattering angles as well. In the case of the few layers of thick graphene sample, like Pelco^®^ graphene 3–5 by Ted Pella (in the following denominated briefly P-graphene), individual graphene layers were rotated above each other by a random angle, resulting in approximately even intensity distribution along the *hk* diffraction rings. As the thickness of five layers of graphene still does not exceed 2 nm, the mean free path of electrons at 200 keV in graphite is ca 110 nm [25], and no multiple scattering is expected. Thus, the size and strain contribution to the diffraction peak shape, as well as its dynamical effect, can be neglected; so, P-graphene can be used as a calibration sample for instrumental broadening. The peak shape of the integrated intensity profile of the few layers of thick graphene sample can be considered as the instrumental broadening was caused by the applied combination of instrumental parameters like TEM lens currents and electron gun settings (acceleration voltage, Wehnelt bias (GunLens parameter in Themis)).

**Figure 2:**
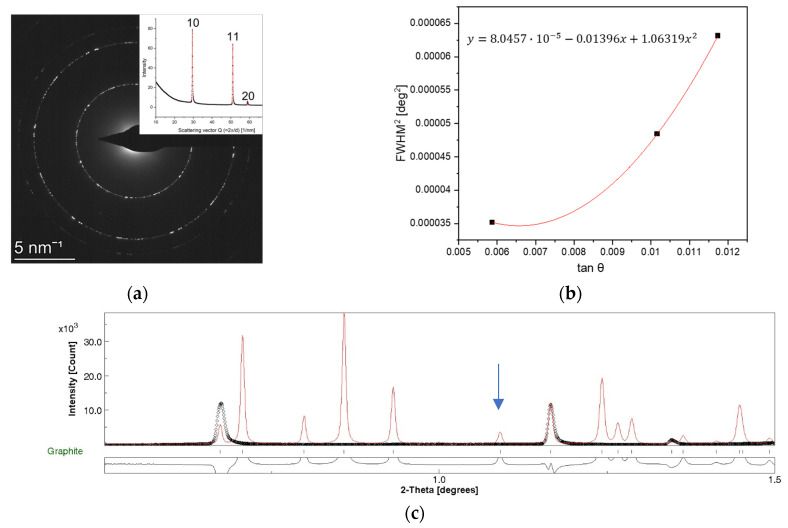
SAED pattern taken from Pelco^®^ graphene 3–5 support film (Ted Pella) with the corresponding integrated intensity profile in the insert (**a**). The solid red line in the intensity profile shows the pseudo-Voigt fit of the diffraction peaks. (**b**) Polynomial fit to obtain the U, V, W Caglioti parameters from the SAED pattern of P-graphene. The error of tan θ is practically equivalent to Δ(sin θ) and calculated using Equation (6). (**c**) Experimental intensity curve of P-graphene sample (black dots) and calculated intensity curve of graphite plotted using the obtained Caglioti (U, V, W) and Gaussianity peak shape parameters (red line). Intensity was scaled to graphene 11, i.e., the graphite 110 peak indicated by an arrow.

Figure 2a shows the SAED pattern taken from P-graphene, which was used as a reference sample for instrumental broadening determination. Three complete diffraction rings, namely the 10, 11, and 20 rings at 2.13, 1.23, and 1.06 Å, respectively, were recorded at the applied camera length. The FWHM values determined by the pseudo-Voigt fitting of the diffraction peaks were around 0.003°, which accounted for a broadening of ±0.01 Å with respect to the peak maximum in terms of interplanar spacing. A similar value was provided by Ref. [26] for the broadening of single crystal diffraction of a few layers of graphene.

To model FWHM as a function of the scattering angle θ, we used the Caglioti function [27].

(4) $${FWHM}^{2}=U\tan^{2}\theta +V\tan\theta +W$$

From the electron diffraction measurement of P-graphene (Figure 2a), three FWHM values were obtained (Table 1), which allowed the determination of the U, V, and W Caglioti parameters (Figure 2b).

From the insert of Figure 2a, it can be seen that the diffraction peaks exhibited slight asymmetric broadening towards higher scattering angles. Asymmetry can be quantified as

(5) $$A=\frac{\left(Q1+Q2\right)-2Q}{2Q}$$

where Q is the scattering vector length at the peak maximum, and Q1 and Q2 are the corresponding Q values at half maximum (at Q ± *FWHM*) on the lower and higher wing of the peak, respectively. Using Equation (5), asymmetry values of the order 10^−4^ were determined.

The asymmetric broadening of diffraction peaks is known from the XRD of random layer materials like smectites [28] or carbon black [29]. Warren [29] explains asymmetric broadening with the diffraction pattern of a two-dimensional lattice, taking all orientations in space with equal probability. Due to the very thin nature of the individual layers and their three-dimensional randomness, the *hk* peak position varies as a function of the tilt angle of the layer with respect to the incident beam. The maximum value of the *hk* peak is obtained if the layer is normal to the beam, and the overall displacement of the maximum (Δsinθ), according to [29], is expressed as a function of the L (in-layer) dimension of the diffracting particle:

(6) $$∆(\sin\theta )=\frac{0.16\lambda }{L}.$$

In contrast to carbon black, P-graphene is a truly two-dimensional material without three-dimensional randomness. Monolayer graphene is commonly slightly corrugated in-TEM, which can lead to some in-plane tilt between the adjacent regions inside the 3 μm diffracted area. The lateral dimension of the ripples (L′) can vary from the Ångström scale for single-layer graphene [30] up to several nanometres for bi-layer graphene [26]. Using the estimate of L′ ≤ 25 nm by Meyer et al. [26], Equation (6) provides an upper limit of Δ(sinθ) ≈ 1.6 × 10^−5^ for the displacement of the maximum peak. As P-graphene is multilayer (3–5 layers), the amplitude of the ripples is expected to decrease by interlayer forces. According to [31], the second derivative of the bending energy density—which characterizes the resistance/toughness of multilayer graphene against bending—is three orders of magnitude larger for 4–5 layers than that for monolayer graphene. As the experimental peak broadened with an increasing tilt angle and the overall effect of undulations quickly diminished with the number of layers [26], we considered that the effect of ripples on broadening and, on the displacement of sinθ in our case, was negligible.

In the Caglioti model, the FWHM values of the diffraction peaks are expressed in a double scattering angle (2θ). According to Equation (4), the FWHM^2^ was plotted against tanθ, and the U, V, and W parameters were obtained (Figure 2b). The error of the FWHM^2^ due to peak asymmetry is in the order of 10^−8^, and so it can be neglected.

The goodness of the Caglioti parameters was checked by plotting the experimental integrated intensity profile of P-graphene together with the calculated intensity profile of graphite in Figure 2c using the U, V, and W parameters from Figure 2b. To estimate Gaussianity, an approximate value of G_0_ = 0.6 was used (Table 1). The good agreement of the experimental peak profile and the peak profile reproduced using the U, V, W, and G_0_ parameters (Figure 2c) proved that the obtained peak parameters allowed to model the instrumental broadening caused by the applied measurement setup satisfactorily.

### 3.2. Rietveld Analysis of Nanocrystalline Hematite—Refining G_0_

**Figure 3:**
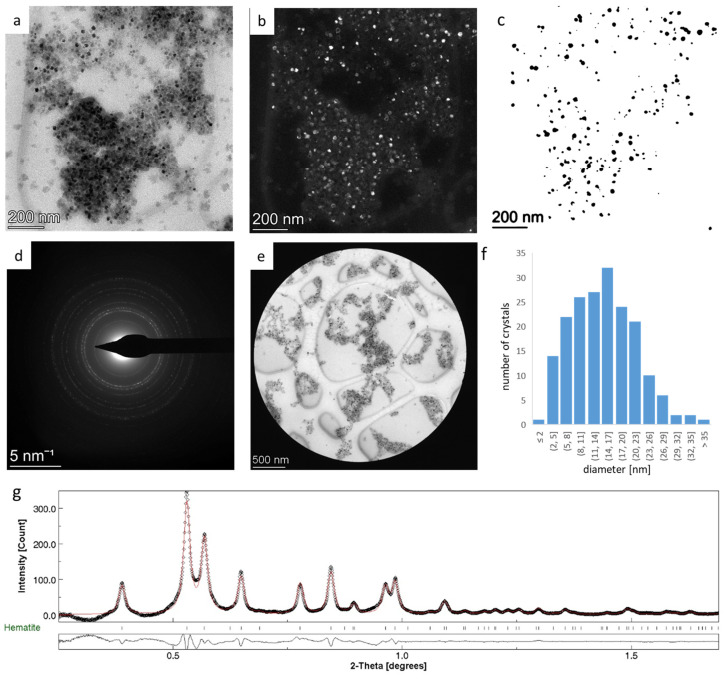
Bright-field (**a**) and corresponding dark-field image (**b**) of hematite nanoparticles. (**c**) Threshold dark-field image used to obtain particle size distribution. (**d**) SAED pattern of hematite nanoparticles and (**e**) the area used to obtain the SAED pattern. (**f**) Crystallite size distribution as determined from (**c**). (**g**) Experimental (black dots) and calculated (red curve) intensity profile. Below the intensity profile, the difference curve between the measurement and fit is plotted.

We applied the obtained peak shape parameters to the Rietveld analysis of monophase homogeneous hematite nanopowder, previously studied with Mössbauer spectroscopy and HRTEM [18]. The aim was to refine the Gaussianity of the instrumental peak profile. Bright-field (BF) and dark-field (DF) images of the hematite nanopowder are presented in Figure 3a,b. Crystallite size distribution (Figure 3f) was obtained from the dark-field image (Figure 3b,c), which resulted in an average crystallite size of 16.2 nm with a standard deviation of 8.4 nm.

The SAED pattern (Figure 3d) was obtained from an area evenly covered by nanoparticles, as indicated in Figure 3e. The absence of texture was checked by the ca. ±17° tilting of the sample holder about the goniometer axis. Camera length calibration and data pre-processing were performed as detailed previously, with the integrated intensity profile used as input data for Rietveld analysis. As data pre-processing included center and ellipticity refinement, instrument parameters such as detector distance (corresponding to the camera length in electron diffraction), center displacement (corresponding to x, y), and tilting error (corresponding to ellipticity parameters α and ε) were excluded from the Rietveld analysis by keeping their values at zero. The background was determined on the intensity profile by interpolation. We used the structure parameters of hematite [32] as initial structure parameters, and instrumental broadening was modeled using the U, V, W, and G_0_ parameters obtained from the SAED pattern of P-graphene.

In the initial stage of the analysis, an approximate value of 0.6 for G_0_ was used based on Table 1, and the angle dependence of Gaussianity (G_1_) was kept at zero. The scale factor was adjusted, and the refinement of basic phase parameters and microstructure parameters was performed while peak shape parameters were kept fixed at the previously determined values. In this stage of refinement, the automated analysis option (“Refinement Wizard”) of the software was used. The refinement cycle was stable and converged fast, resulting in an average crystallite size of 14.47 nm. In the last step of the automated analysis, the G_0_ and G_1_ parameters were also included in the refinement. The peak shape parameters used in the final refinement cycle are listed in Table 2. It is important to note that this step did not produce any significant change in lattice parameters, microstructure parameters, and R-values (Table S1).

The experimental and calculated diffraction profiles and the difference curve are displayed in Figure 3g, and the detailed results of the analysis are summarized in Table S1.

### 3.3. Rietveld Analysis of Cu-Ni Thin Films—Crystallite Size and Phase Ratio from Overlapping Rings

Two-component nanocrystalline Cu-Ni thin films were deposited at RT and 150 °C and measured by SAED. Integrated intensity profiles were analyzed using the Rietveld method. Cu and Ni are isostructural (Fm-3m) with a_0_ lattice parameters of 3.61496 Å (AMCSD-0011145) and 3.52387 Å (AMCSD-0011153), respectively (2.5% difference). Our purpose was to extract the phase fraction and crystallite size of the two phases from SAED using the experimentally determined peak shape parameters. Control measurements of phase fraction were performed by TEM-EDS exactly at the position of the SAED measurement. The crystallite size difference between the two phases was recognized by the visual observation of the SAED patterns.

**Figure 4:**
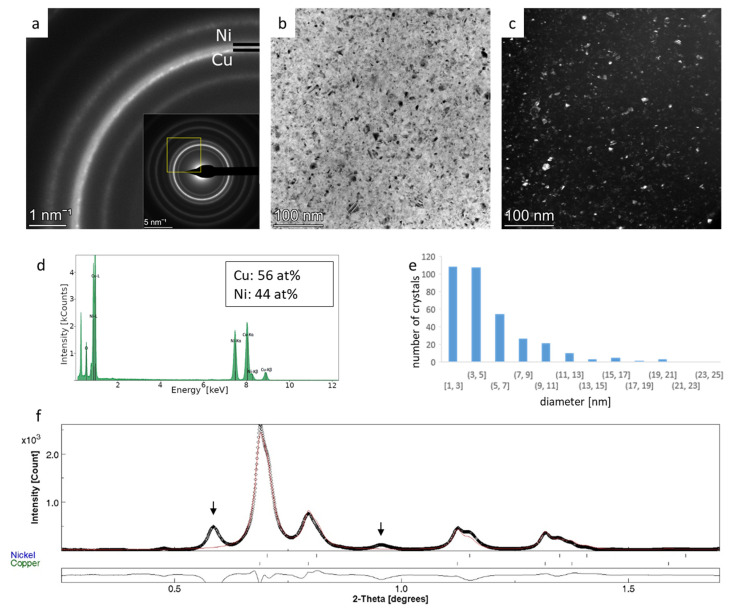
SAED pattern of Cu-Ni thin film deposited at room temperature; Cu and Ni 111 diffraction rings are marked (**a**). The yellow square in the inset indicates the zoomed region. Bright-field (**b**) and dark-field images (**c**), EDS spectrum (**d**), and crystallite size distribution determined based on DF image (**e**). The integrated intensity profile (black dots) and fitted profile after final refinement are plotted on (**f**). Below the diagram, the difference curve between measurement and fit is seen. Arrows indicate Cu-oxide rings.

**Figure 5:**
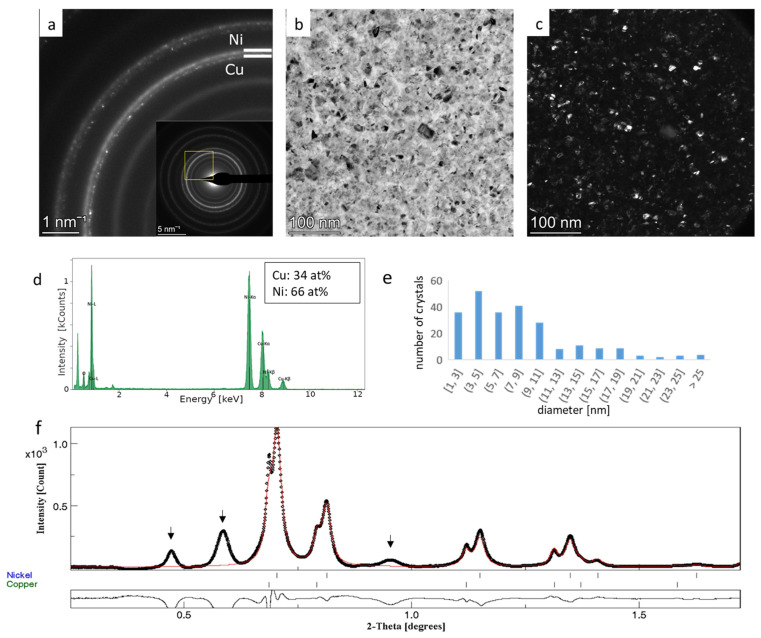
SAED pattern of Cu-Ni thin film deposited at 150 °C (**a**); Cu and Ni 111 are marked. The yellow square indicates an enlarged region, bright-field (**b**) and dark-field image (**c**), EDS spectrum (**d**), and crystallite size distribution determined based on the DF image (**e**). Integrated intensity profile (black dots) and fitted profile after final refinement (**f**). Below the diagram, the difference curve between the measurement and fit is seen. Arrows indicate Cu-oxide rings.

Copper tends to oxidize fast; thus, the formation of an amorphous surface oxide layer cannot be avoided, as indicated by the presence of broad rings marked by arrows in Figure 4f and Figure 5f. These rings are well separated from the metal components, so they could be excluded from the analysis easily. The diffraction rings of Cu and Ni strongly overlap as they are broadened due to the few nanometers of crystallite size (Figure 4 and Figure 5). Careful observation of the SAED patterns (Figure 4a and Figure 5a) reveals that the inner arc of each ring corresponding to larger interplanar spacing values is sharper and exhibits a slightly spotty character, while the outer arc is more diffuse, suggesting that the crystallite size of copper is larger than that of nickel. The separation of Cu and Ni rings is more enhanced in the case of the 150 °C sample (Figure 5a) due to the formation of larger grain sizes at higher temperatures. Crystallite size distribution was determined by processing DF images (Figure 4c and Figure 5c), and the average crystallite size of 5.1 nm and 8.2 nm were obtained for RT and 150 °C samples, respectively. These average crystallite sizes include both Cu and Ni crystallites. The increase in the average value is in agreement with expectations; however, the distinction between the size of Cu and Ni nanocrystals cannot be made (Figure 4e and Figure 5e).

In the case of the RT sample, the automated analysis did not converge; thus, the basic phase parameters were first refined with a fixed atomic ratio (50–50%), which was followed by the separate refinement of microstructure parameters. The procedure was repeated several times to check the reproducibility of the results and each time for at least 15 iterations needed to reach convergent cycles. The phase ratios and crystallite sizes for the two components resulted in 58.4 at% and 8.23 nm for Cu, and 41.6 at% and 5.44 nm for Ni, respectively. The composition measured with EDS is 56 at% Cu and 44 at% Ni (Figure 4d). In the case of the 150 °C film, the same automated strategy was used successfully as in the case of hematite nanoparticles, and the basic refinement yielded phase ratios and crystallite sizes of 30.7 at% and 25.3 nm for Cu and 69.3 at% and 10.9 nm for Ni, respectively. The composition measured with EDS was 34 at% Cu and 66 at% Ni (Figure 5d). The obtained results are detailed in Table S2 in the “Basic refinement” column.

**Figure 6:**
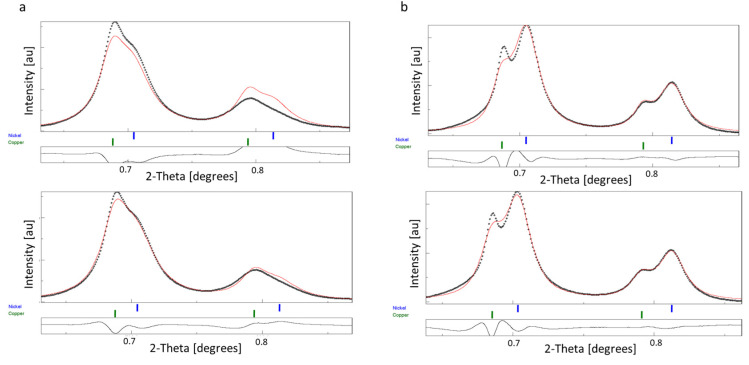
**Peaks** 111 and 200 of Cu-Ni RT (**a**) and Cu-Ni 150 °C; (**b**) thin films after basic (**top row**) and final refinement (**bottom row**). Note the deviations between measured (black) and fitted (red) curves. In the case of the RT sample, intensity ratios indicate the 111 preferred orientation.

The careful observation of intensity ratios of 111 and 200 peaks revealed a smaller deviation between the fitted and measured intensities (Figure 6). In the case of the RT sample (Figure 6a), the intensity ratio of the 111 and 200 diffraction peaks indicated the development of the preferred <111> orientation of nanocrystals. To include the texture parameter in the Rietveld analysis, we applied the March–Dollase approach [33], which allowed us to specify the crystallographic direction of the preferred orientation. The goodness of fit has improved apparently without a notable change in crystallite size and atomic ratio values. The obtained March parameters (0.71 and 0.67 for Cu and Ni, respectively) indicated a small degree of preferred orientation.

In the case of the 150 °C sample, there was no clear indication of preferred orientation (Figure 6b). Thus, after basic refinement, the “arbitrary texture” option was activated, which allowed the variation in intensity ratios without crystallographic constraints [34,35]. This option aims to modify intensities freely to reach the best fit between the observed and modeled values. No noticeable improvement was observed visually (Figure 6b), and phase ratios and crystallite sizes for the two components resulted in 35.8 at% and 17.1 nm for Cu and 64.2 at% and 11.4 nm for Ni, respectively (Table S2), which falls closer to the composition measured with EDS (Figure 5d).

## 4. Discussion

### 4.1. Validation of the Peak Broadening Determination of TEM

The instrumental broadening of TEM in the whole scattering angle range was modeled with the Caglioti relation [4], using the FWHM values of the SAED of a few layers of thick turbostratic graphene (P-graphene) foil. The successful application of the automated refinement algorithm on SAED patterns of hematite nanopowder and nanocrystalline CuNi thin films proved the convenience of the instrumental broadening function. An analysis of single-phase hematite nanopowder allowed the refinement of the Gaussianity parameter as well. Crystallite sizes obtained from the refinements agreed well with image processing data.

During the analysis, our approach was to reduce the number of refined parameters as much as possible. By applying standard acquisition conditions, the variation in camera length was below 0.1% [3], so the correction for detector distance/camera length could be skipped. Data pre-processing before obtaining the integrated intensity profile allowed the diffraction pattern center to be found and corrected ellipticity with high accuracy [20]; thus, no further refinement of the center displacement or tilt error was needed. The Caglioti parameters U, V, and W for peak shape modeling were taken from the calibration measurement and kept fixed during the whole procedure. The only instrument-related refined parameters were G_0_ and G_1_. No direct information on the scattering angle dependence of Gaussianity (G_1_) was obtained during the calibration measurement. The measured G_0_ of the diffraction peaks of the P-graphene standard varied in the range of 0.56–0.72. When initiating the Rietveld analysis, an approximate starting value for G_0_ (0.6) and zero for G_1_ were applied. Then, as a final step of the Rietveld analysis of hematite nanopowder, the refinement of the G parameters was performed, which resulted in 0.64 and 0.02 values for G_0_ and G_1_, respectively. These values show minor changes with respect to the starting values.

It is remarkable that the variation in G parameters with respect to the starting values did not notably improve the R-factors; significant changes were neither induced in the lattice parameter nor crystallite size data (Table S1). It indicates that after complete TEM alignment, the Caglioti modeling of peak broadening with an approximate G_0_ value obtained from the pseudo-Voigt fitting of diffraction peaks and neglected G_1_ provides a satisfactory peak shape model at the applied experimental conditions. Thus, we were able to extract crystallite size information from the diffraction peak profile by a quick routine procedure. Moreover, it also demonstrates that the Rietveld analysis of the standardized electron diffraction measurement of an appropriate nanopowder provides G_0_ and G_1_ parameters for a specific lens current combination. These G values can be used later during the Rietveld analysis of more complicated samples.

In contrast to nanopowders, the determination of the average crystallite size of thin film samples is not straightforward using image processing techniques. If the film thickness exceeds the average crystallite size, overlapping grains occur, which hampers thresholding dark-field images. Also, strain fields, bending contours, or the moiré effect may contribute to the contrast of DF images, which prevents the effective use of automated image analysis routines [36]. Moreover, on a DF image, only a small range of crystal orientations was present, which may distort crystallite size data, particularly in the case of some degree of preferred orientation of nanocrystals or anisotropic shape. These factors make crystal size determination by image processing more complex and increase the uncertainty of the results. In the case of multiphase nanocrystalline thin films, the rings of the component phases can be so close to each other that their separate use for DF image formation is not possible. In this case, crystallite size analysis based on dark-field images does not make a difference between the two components.

Rietveld analysis of SAED patterns of binary Cu-Ni thin films was applied to analyze the assumed crystallite size difference between the two components with broad overlapping reflections. In the case of both RT and 150 °C samples, results provided larger values for Cu than for Ni, which verified our presumption based on the visual observation of SAED patterns. We have to note that the crystallite size distribution histogram of the CuNi RT sample based on DF image processing (Figure 4e) exhibits a single maximum, which means that the average size difference between the two components cannot be resolved. In the case of the 150 °C sample, the histogram exhibits a minor peak around 17 nm (Figure 5e), which corresponds to a second population; however, the crystalline phase of the two populations cannot be recognized in such histograms. Rietveld analysis allowed the separate determination of the crystallite sizes of the two phases simultaneously in both Cu-Ni thin film samples. DF images can provide reasonable initial values for the Rietveld refinement and can also be used as control data for the refined values.

The development of the preferred orientation of (111) planes in the RT sample has been quantified by applying the March–Dollase approach. After texture parameters were included in the analysis, the R-factors improved without variation in crystallite size and atomic ratio values (Table S2). In the case of the 150 °C sample, the intensity difference between experimental and fitted curves did not indicate a straightforward development of the preferred orientation. Accordingly, the “Arbitrary texture” model resulted in a better fit in terms of R-values, and notable changes in the crystallite size and atomic ratio values with respect to the basic refinement. We conclude from these results that the increase in the copper 111 peak intensity should not be related to preferred orientation. The good intensity match of the measured and fitted copper 200 peaks (Figure 6b) also supports the lack of preferred orientation.

Besides their preferred orientation, several factors can modify the intensity ratios of random orientation distribution. While in powder XRD several thousands of crystallites are measured, at the same time, in SAED, especially when smaller apertures are used, the selected area may contain a lower number of diffracting crystals. In these cases, orientation statistics will be poor, and the condition of randomness is not fulfilled. In thin films with inhomogeneous grain size, larger grains contribute with higher intensity to the SAED due to the larger size of coherently scattering domains. With increasing thickness, the probability of double diffraction increases too. Distorted intensity ratios on the diffraction pattern lead to a poorer fit during Rietveld analysis. Moreover, if the texture is not defined correctly or multiple textures are present, the Rietveld algorithm does not converge. In these cases, the use of the “Arbitrary texture” model can be a good choice to overcome this difficulty. The “Arbitrary texture” model is a robust fitting algorithm that is not sensitive to the source of variation in intensity and is able to improve intensity fit without physical information on the crystallographic texture. For example, Wenk et al. [34] used it to compensate for the coarse nature of their standard. In the case of the Cu-Ni 150 °C sample, we think that the observed 111/200 intensity ratio of the copper phase was due to the strong Bragg reflections of some larger crystallites in the measured area rather than to the preferred orientation. It has to be noted that the atomic ratio obtained using the “Arbitrary texture” model provided the best fit with EDS data. This indicates that forcing a reasonably assumed but incorrect preferred orientation may improve the R-factors of the Rietveld analysis but does not provide real information on the nanostructure, which is an important limitation of electron diffraction-based Rietveld analysis.

### 4.2. Benefits of the Single Step in-TEM Determination of Instrumental Broadening

The determination of instrumental peak broadening is fundamental for diffraction pattern-based nanostructure analysis. In the case of well-resolved peaks, deconvolution can be performed using the measured peak profile of a single crystalline sample. This method was followed by [37] during the grain size analysis of the nanocrystalline FeAl alloy. Alternatively, peak shape modeling as a function of the diffraction angle, e.g., Caglioti modeling, can be performed, which allows the analysis of complex diffraction patterns with peak overlaps, as in the case of lower symmetry and/or multicomponent samples. The procedure proposed in [15] allows the instrumental peak to be obtained, broadening indirectly with the help of complementary XRD measurements. Their method determines the instrumental broadening as the difference between the measured broadening and grain size-related broadening. Our procedure makes the determination of the instrumental broadening of the applied electron optical setup easier, as no additional measurements are needed, and peak broadening parameters can be obtained from a single measurement on an appropriate calibration sample. Moreover, our proposed approach offers a direct measurement of the instrumental broadening, which allows for a higher accuracy compared to the indirect method in [15].

Our calibration sample was a few layers of thick graphene, which is available commercially; no additional synthesis procedures were needed, and this material could be stored at room conditions without alteration for a long time. Graphene covers the TEM grid uniformly, while nanoparticle calibration samples may form thicker aggregates, which leads to enhanced multiple scattering. Moreover, graphene foil is self-supporting; thus, an amorphous background on the SAED pattern is avoided. These factors contribute to a reduced background on the diffraction profile. P-graphene can also be used as supporting foil in the case of an electron diffraction measurement of a nanoparticle sample, serving at the same time as an internal standard both for the determination of camera length and peak broadening parameters.

The determination of peak broadening parameters of the TEM using the SAED of an appropriate calibration sample allows a reliable separation of instrumental and sample contribution to peak width on the analyzed specimen. Then, during Rietveld analysis, the instrumental parameters can be kept at previously determined fixed values. In contrast to XRD, it is an essential issue in electron diffraction because of the wide variety of available lens current combinations, some of which produce qualitatively similar SAEDs with clearly different instrumental contributions [38]. A systematic study of the effect of the camera length and selected area aperture on peak width has been published recently [17]; however, the effect of factors like beam convergence and accelerating voltage also needs to be considered. Additionally, the Modulation Transfer Function of the recording medium [38,39,40] may also affect the width of the diffraction rings; thus, the application of the highest available camera resolution is reasonable.

By separating the determination of instrumental parameters from the analysis of sample parameters, we aim to ensure that the applied instrumental parameters have a physical meaning (i.e., they are related to a certain, intentionally chosen combination of lens currents), and we intend to avoid their variation for better mathematical fit and better R-factors without physical meaning. This approach guarantees an improved accuracy/reliability of nanostructure parameters for the analyzed nanomaterial. In addition, as TEM provides supplementary information to SAED, like DF imaging and/or EDS measurements, the obtained sample parameters can generally be validated to some extent.

The correct alignment of the TEM ensures not only calibration accuracy and the reproducibility of camera length but also the minimization and steadiness of instrumental peak broadening. We expect that the combination of the standard acquisition procedure [3] with the in-TEM determination of instrumental broadening will promote a more reliable and sophisticated analysis of nanostructure parameters of complex nanomaterials, like the preferred orientation of multicomponent thin films or anisotropic crystallite size in biological nanomaterials.

## 5. Conclusions

The single step in-TEM method proposed in this paper allows the direct determination of the instrumental broadening of the TEM from one electron diffraction measurement without the need for an additional XRD facility. This method provides more accurate broadening parameters compared to indirect methods, which determine instrumental broadening as the difference between the measured broadening and the grain size-related broadening. The single step in-TEM method requires an appropriate calibration sample, e.g., a few layers of commercially available self-supporting thick graphene foil. The diffraction peak shape is modeled as a function of the scattering angle using the Caglioti relation and can be directly applied during Rietveld analysis. Considering the different possible lens current combinations during electron diffraction measurements, the parameters of instrumental broadening should be determined for each applied lens current combination, even if using the same TEM. In this way, instrumental broadening parameters can be handled separately from sample-related broadening effects, which contributes to the higher reliability of nanostructure parameters.

The Rietveld analysis of hematite nanopowder and two-component nanocrystalline Cu-Ni thin films support the fact that correctly determined peak shape parameters provide a reliable model for the instrumental peak broadening of the TEM. By applying standard acquisition conditions and performing proper data pre-processing, instrumental broadening can be kept steady. This makes the automated refinement procedure very effective in extracting average and statistically representative information on the nanostructure from peak shape by the simple and fast evaluation of electron diffraction measurements.

The single-step in-TEM procedure for the determination of instrumental broadening is most beneficial in the case of complex electron diffraction patterns of multicomponent and low symmetry nanostructured materials, and is expected to successfully manage morphological anisotropy as well.

## Figures and Tables

**Table 1:** FWHM and Gaussian parameters of P-graphene diffraction peaks obtained with pseudo-Voigt fitting.

	Peak 10	Peak 11	Peak 20
FWHM [nm^−1^]	0.26	0.306	0.348
Gaussianity, G_0_	0.62	0.56	0.72

**Table 2:** Caglioti parameters (in deg^2^) and Gaussian parameters used in the final refinement cycle of hematite nanopowder. The Caglioti parameters were obtained experimentally from P-graphene and the Gaussian parameters were refined.

U	1.06319
V	−0.01396
W	8.0457·10^−5^
G_0_	0.64377
G_1_	0.02128

## Data Availability

Measurement data are available upon request.

## Supplementary Materials

The following supporting information can be downloaded at: https://www.mdpi.com/article/10.3390/nano14050444/s1, Table S1: Summary of the results of Rietveld analysis obtained for nanocrystalline hematite Table S2: Lattice parameters and crystallite size data of Cu-Ni thin films as obtained after basic and final refinement cycle.
