# Supplementary material for: Improved Method for Electron Powder Diffraction-Based Rietveld Analysis of Nanomaterials
Source: Nanomaterials (Basel). 2024 Feb 28;14(5):444. doi: 10.3390/nano14050444 (PMC10934415; doi:10.3390/nano14050444)
Supplement: Supplementary file 1 [file nanomaterials-14-00444-s001.zip › nanomaterials-2877167-supplementary.pdf]

Supplementary information

Improved Method for Electron Powder Diffraction-Based Rietveld Analysis of Nanomaterials

Viktória K. Kis [1,2,*], Zsolt Kovács [3] and Zsolt Czigány [1,*]

[1] HUN-REN Centre for Energy Research, Institute of Technical Physics and Materials Science, Konkoly-Thege Miklós út 29-33, H-1121 Budapest, Hungary
[2] Department of Mineralogy, Eötvös Loránd University, Pázmány Péter sétány 1/c, H-1117 Budapest, Hungary
[3] Department of Materials Physics, Eötvös Loránd University, Pázmány Péter sétány 1/a, H-1117 Budapest, Hungary; kovacs.zsolt@ttk.elte.hu
* Correspondence: kis.viktoria@ek.hun-ren.hu (V.K.K.); czigany.zsolt@ek.hun-ren.hu (Z.C.)

Table S1: Summary of the results of Rietveld analysis obtained for nanocrystalline hematite

	Results of automated analysis with G0 = 0.6 and G1 = 0	Results of automated analysis with refined G0 and G1
a_0 [Å]	5.0366(3)	5.0386(1)
c_0 [Å]	13.751(6)	13.758(2)
Crystallite size [nm]	14.47	14.81
microstrain	$8.1 \cdot 10^{-5}$	$2.8 \cdot 10^{-6}$
R_{wp} [%]	1.66	1.49
R_{exp} [%]	5.51	5.51
R_B [%]	1.14	1.05

Table S2: Lattice parameters and crystallite size data of Cu-Ni thin films as obtained after basic and final refinement cycle.

	Cu-Ni thin film deposited at RT			Cu-Ni thin film deposited at 150 °C		
	Basic refinement	Refinement with arbitrary texture	Refinement with M-D texture	Basic refinement	Refinement with arbitrary texture	Refinement with M-D texture
Cu a_0 [Å]	3.6084(4)	3.6165(4)	3.6081(9)	3.6142(6)	3.6193(7)	3.6167(3)
Cu crystallite size [nm]	8.23	9.5	8.23	25.3	17.1	23.4
Cu microstrain	$1.6 \cdot 10^{-3}$	$3.5 \cdot 10^{-3}$	$1.6 \cdot 10^{-3}$	$4.2 \cdot 10^{-4}$	$7.3 \cdot 10^{-5}$	$1.9 \cdot 10^{-4}$
Cu at%	58.4	60.1	60.4	30.7	35.8	31.6
Cu (111) M-D param			0.71			0.77
Ni a_0 [Å]	3.5239(3)	3.5310(1)	3.5236(3)	3.5192(5)	3.5238(9)	3.5219(9)
Ni crystallite size [nm]	5.44	6.11	5.44	10.9	11.4	11.7
Ni microstrain	$3.5 \cdot 10^{-3}$	$5.6 \cdot 10^{-3}$	$3.4 \cdot 10^{-3}$	$1.9 \cdot 10^{-4}$	$4.5 \cdot 10^{-5}$	$3.8 \cdot 10^{-5}$
Ni at%	41.6	39.9	39.6	69.3	64.2	68.4
Ni (111) M-D param			0.67			1
R_{wp} [%]	6.80	2.01	4.27	3.24	2.5	3.21
R_{exp} [%]	4.30	4.31	4.13	6.07	6.08	6.07
R_B [%]	5.11	3.06	4.19	4.29	3.95	4.28
